# Tacrolimus-Associated Tremor in Renal Transplant Patients: Potential Impact of the Galenic Formulation

**DOI:** 10.3390/ph18101488

**Published:** 2025-10-03

**Authors:** Jordi Rovira, Olga Millán, Pedro Ventura-Aguiar, Mercè Brunet, Fritz Diekmann

**Affiliations:** 1 Nephrology and Transplantation (LENIT), Fundació Recerca Clínic Barcelona-Institut d’Investigacions Biomèdiques August Pi i Sunyer (FRCB-IDIBAPS), 08036 Barcelona, Spain; jrovira1@recerca.clinic.cat (J.R.); pventura@clinic.cat (P.V.-A.); 2 RICORS(RD21-0005-0003), Instituto de Salud Carlos III, 28029 Madrid, Spain; 3 Pharmacology and Toxicology Section, Centre de Diagnòstic Biomèdic (CDB), Hospital Clinic of Barcelona, FRCB-IDIBAPS, University of Barcelona, 08036 Barcelona, Spain; omillan@clinic.cat; 4 Biomedical Research Center in Hepatic and Digestive Diseases (CIBEREHD), Instituto de Salud Carlos III, 28029 Madrid, Spain; 5 Department of Nephrology and Kidney Transplantation, Institut Clínic de Nefrologia I Urologia (ICNU), Hospital Clínic de Barcelona, 08036 Barcelona, Spain

**Keywords:** kidney transplantation, tremors, calcineurin inhibitors, bioavailability, wearable wireless sensor, immunological biomarkers

## Abstract

**Background/Objectives:** Tacrolimus is the most used immunosuppressive agent in solid organ transplantation due to its efficacy in preventing acute rejection, but it has a narrow therapeutic range, and overexposure often leads to toxicities, including neurological side effects like tremors. Tremor affects up to 54% of renal transplant patients under tacrolimus. Extended-release tacrolimus (LCPT) has demonstrated efficacy in reducing tremor severity, as evidenced by studies employing quality of life (QoL) questionnaires, the Fahn–Tolosa–Marin (FTM) scale, and Accelerometer devices. The objectives of this study were to evaluate the benefits of the conversion to LCPT formulation in kidney transplant recipients experiencing tremors on prolonged-release tacrolimus (PR-TAC) treatment and to validate the DyCare device, a wearable wireless sensor for tremors. **Results:** The DyCare device measured tremor frequencies of 8.74 ± 0.11 Hz and 1.36 ± 0.08° and 17.38 ± 1.16°, as root mean square (RMSx100 for accelerometer and Gyroscope, respectively) in PR-TAC patients. After switching ten patients to LCPT, tremor severity significantly decreased, as confirmed by DyCare and the QoL in the Essential Tremor Questionnaire (QUEST). Additionally, LCPT allowed a 34% reduction in tacrolimus dosage while maintaining therapeutic trough concentrations. Immunological and pharmacodynamic biomarkers (p-miR-210-3p, p-IL10, p-IL12p70, p-IFNγ uCXCL10, NFAT-regulated gene expression) confirmed stable immunosuppression post-conversion. **Conclusions:** The conversion to the LCPT formulation significantly reduced tremors in kidney transplant recipients without altering their immunological status, as confirmed through a panel of immunologic and pharmacodynamic biomarkers. The DyCare device enables a precise quantification of tremors in transplant recipients, allowing physicians to optimize treatment strategies.

## 1. Introduction

Calcineurin inhibitors are widely used immunosuppressants in contemporary organ transplantation. In particular, tacrolimus is the most used due to its effectiveness in preventing rejection [1]. Tacrolimus has a restricted therapeutic range and displays considerable inter- and intraindividual variability in pharmacokinetics and clinical response [2,3]. Currently, three different tacrolimus formulations are available with a specific pharmacokinetics profile: twice-daily immediate-release tacrolimus (IR-TAC), once-daily prolonged-release tacrolimus (PR-TAC), and once-daily extended-release (MeltDose^®^) tacrolimus (LCPT) [4]. The increased tacrolimus bioavailability from LCPT is associated with a significant lower Cmax [5] with a consistent tacrolimus exposure (AUC) at an approximately 30–36% lower dose compared to twice-daily (IR-TAC)^2,3^ or 30% compared to PR-TAC [4], showing non-inferior efficacy and similar safety compared with other tacrolimus formulations [6,7]. In order to achieve optimal efficiency and minimize dose-related toxicity, tacrolimus treatment should be adjusted individually according to drug trough blood levels [8,9]. Recently the pre-dose tacrolimus concentration-to-daily-dose (C_0_/D) ratio has also been proposed as a prognostic marker for poor outcome, identifying fast metabolizers, which are the patients at high risk of developing toxicities or rejection [10,11]. Despite the close monitoring of patients which is standard practice today, several neurologic tacrolimus-induced adverse effects (AEs) have been reported at peak whole blood tacrolimus blood concentrations [12]. Concretely, tremor occurs in 34–54% of renal transplant patients under tacrolimus treatment [13]. Tremor is associated with a significant decrease in the quality of life (QoL) of transplant patients [14,15]. Furthermore, inefficacy of immunosuppressant drugs is associated with non-adherence to medication regimens in transplant patients [14,16,17].

The STRATO study was the first study conducted in kidney transplant recipients (KTR) to evaluate the impact of tacrolimus formulation conversion from IR-TAC to LCPT on tremors experienced by KTR [18]. Langone et al. showed that LCPT was associated with a clinically meaningful improvement of hand tremor, leading to a better quality of life [18]. Unfortunately, the study was focused on a short-term analysis after 7 days.

Recently, a non-interventional, non-randomized, observational ELIT study revealed that all patients had a significant improvement of tremor due to QoL questionnaires and ratio C_0_/D post-switch to LCPT irrespective of the previous tacrolimus formulation administered (IR-TAC or PR-TAC) [19]. Recently, several promising non-invasive biomarkers have been identified to enhance and personalize tacrolimus treatment [11,20].

**Figure 1:**
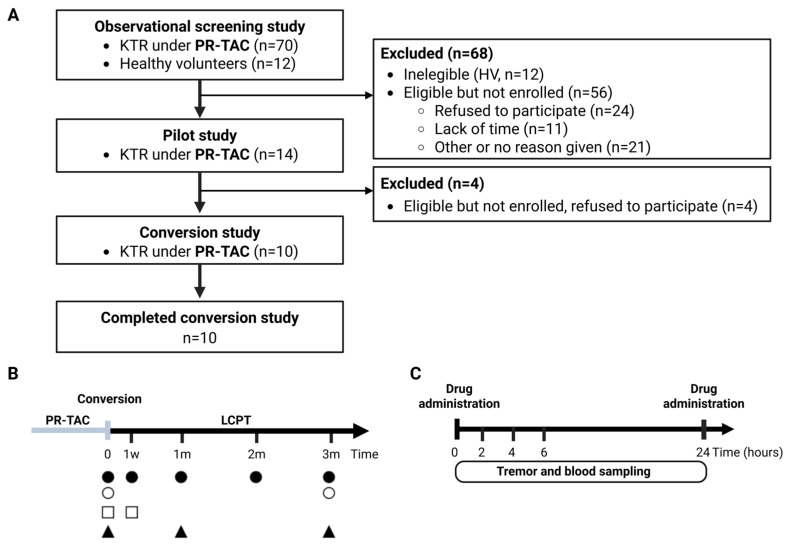
**Experimental design.** (**A**) The STROBE flowchart. (**B**) Conversion study protocol. (**C**) Area under the curve (AUC), time points required for tacrolimus AUC and Tremor AUC. KTR, kidney transplant recipients; PR-TAC, prolonged release-tacrolimus; LCPT, extended-release tacrolimus; w, week; m, months; ●, tremor evaluation by DyCare device; O, tremor evaluation by QUEST; □, tacrolimus AUC; ▲, biomarkers. Created by BioRender (Toronto, ON, Canada).

In this study, we conducted a conversion trial in ten adult kidney transplant patients under PR-TAC treatment to evaluate the reduction in tacrolimus tremor using LCPT formulation (Figure 1). In addition, we have standardized and evaluated the use of the DyCare device, a wearable wireless sensor designed to measure tremors during post-transplant follow-up visits, to identify patients with tremors associated with tacrolimus treatment, in the total number of patients who underwent testing, as well as in the cohort of the conversion. In addition, we have evaluated the impact of this conversion on tacrolimus dose, AUC, C_0_, C_0_/D, and pharmacodynamic and immunological biomarkers of immunosuppressive activity as well as immunological response.

## 2. Results

### 2.1. Observational Screening Study

**Figure 2:**
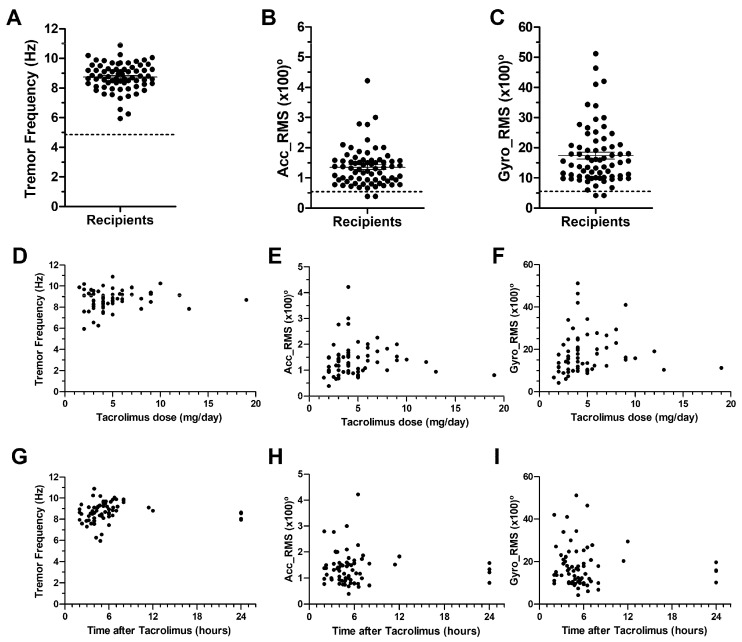
**Evaluation of tremors in kidney transplant recipients using DyCare device.** (**A**) Tremor Frequency. (**B**) Accelerometer RMS. (**C**) Gyroscope RMS. (**D**) Impact of tacrolimus dose on Tremor Frequency. (**E**) Impact of tacrolimus dose on Accelerometer RMS. (**F**) Impact of tacrolimus dose on Gyroscope RMS. (**G**) Impact of Time after tacrolimus administration on Tremor Frequency. (**H**) Impact of Time after tacrolimus administration on Accelerometer RMS. (**I**) Impact of Time after tacrolimus administration on Gyroscope RMS. The line that appears in A,B and C indicates the mean value for each parameter in the healthy volunteer cohort.

**Figure 3:**
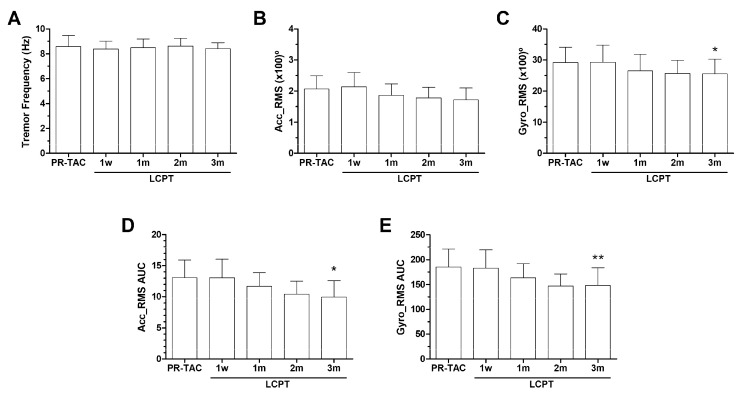
**Impact of tacrolimus conversion on tremors using DyCare Device.** (**A**) Tremor Frequency at baseline over time. (**B**) Tremor Accelerometer at baseline over time. (**C**) Tremor Gyroscope at baseline over time. (**D**) Tremor Accelerometer AUC over time. (**E**) Tremor Gyroscope AUC over time. Acc, Accelerometer; AUC, area under the curve; Gyro, Gyroscope; RMS, root mean square; PR-TAC, prolonged release-tacrolimus; LCPT, extended- release tacrolimus; w, week; m, months. Significantly different when compared to PR-TAC (* *p* < 0.05; ** *p* < 0.01).

A total of 70 kidney transplant recipients and 12 healthy volunteers were included in the Observational Screening study (Table 1). The Tremor Frequency observed in renal transplant patients receiving tacrolimus (PR-TAC) was higher than in healthy volunteers. Kidney transplant recipients showed 2.5 to 3 times higher Accelerometer RMS and Gyroscope RMS than healthy volunteers (Figure 2). We have established that patients under PR-TAC treatment that experienced tremors in our center had a Frequency > 6.1 HZ, Acc_RMS > 0.785°, and Gyro_RMS > 7.59° with a sensitivity > 85% and specificity > 90% (Figure S2). No statistically significant correlation was identified between the dosage of tacrolimus ingested and any of the data generated with the DyCare device (frequency, Acc_RMS, and Gyro_RMS) (Figure 3D–E). Similarly, no correlation was found between the time elapsed since the last tacrolimus intake and the tremor parameters (Figure 2G–I).

### 2.2. Pilot Study. Tremor, and Tacrolimus Blood Level Correlations

In addition, we studied the correlation between tacrolimus blood levels and tremor parameters in a set of 14 recipients of kidney transplants at different time points (pre-dose, after 2, 4, and 6 h). Our data revealed no linear correlation between parameters of tremor and tacrolimus blood levels (Figure S3).

### 2.3. Conversion Study

#### 2.3.1. Patient Characteristics

The patients included in the conversion study (PR-TAC to LCPT-TAC) were predominantly male (70%) with a mean age of 59.4 ± 12.4 years. Patients had received a renal graft 3.9 ± 2.5 years prior from living donors (80% of cases). Hypertension was present in 90% of patients, and dyslipidaemia in 40%. The renal function and general condition before conversion and at the end of the study are shown in Table 2.

#### 2.3.2. Impact of Conversion on Tremor

*Tremor evaluation using DyCare device.* Figure 3 shows the most significant results obtained with the DyCare device. First, the evaluation of the tremor at baseline, before drug administration at different time points of the study, showed that the tacrolimus conversion reduced Gyro_RMS after 3 months of conversion (Figure 3A–C). Then, we calculated the tremor experienced over 6 h using area under the curve (AUC) at 1 week, 1 month, 2 months, and 3 months. The reduction in both Acc_RMS AUC and Gyro_RMS AUC was found to be statistically significant at the 3-month follow-up, post-conversion (Figure 3D,E).

**Figure 4:**
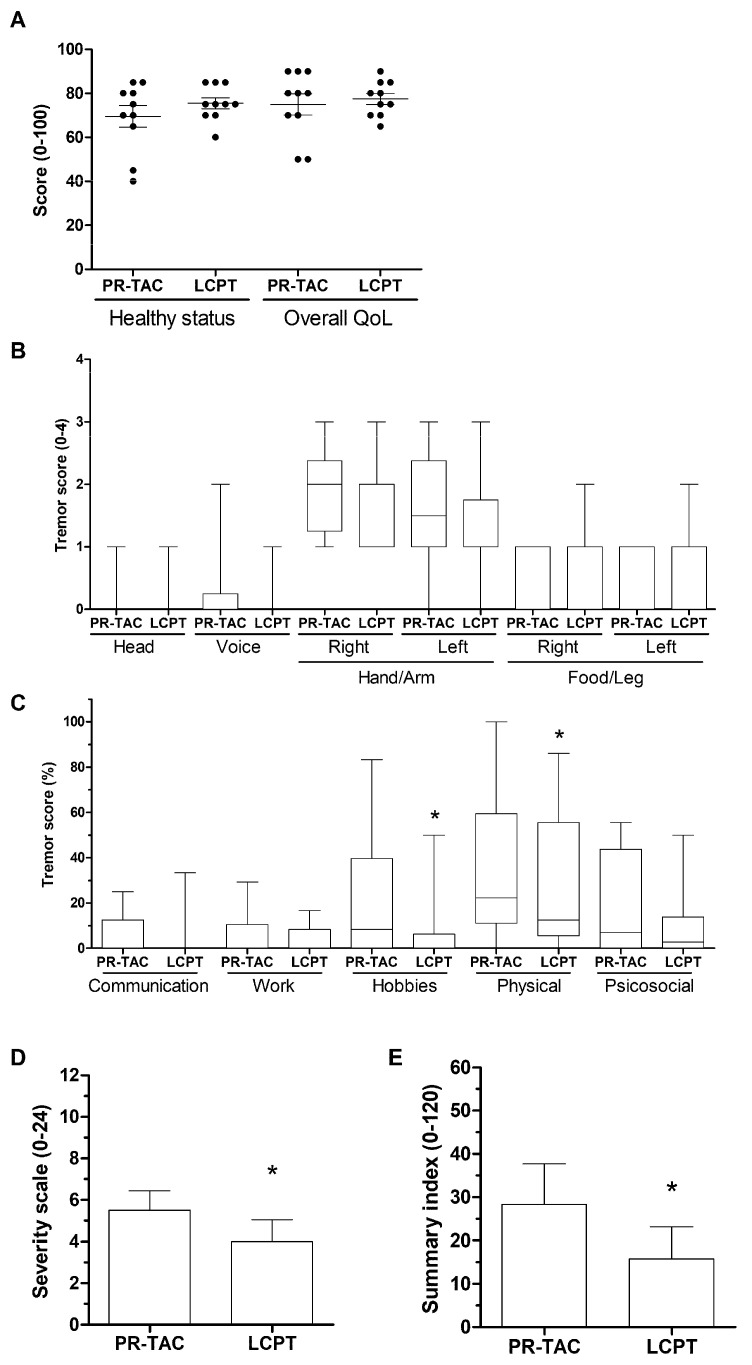
**Evaluation of tremor in kidney transplant recipients using QUEST.** (**A**) Impact of tacrolimus conversion on healthy status and overall quality of life. (**B**) Impact of tacrolimus conversion on tremor severity detected in six body parts. (**C**) Impact of tacrolimus conversion on tremor was analyzed according to the five life dimensions described in QUEST. (**D**) Impact of tacrolimus conversion on the severity scale. The severity scale is the summatory of tremor score (0–4) for all items described in Figure 4B. (**E**) Impact of tacrolimus conversion on the summary index. The summary index is the summatory of tremor score (0–4) for all 30 items included in Figure 4C. PR-TAC, prolonged release-tacrolimus; LCPT, extended-release tacrolimus. Significantly different when compared to PR-TAC (* *p* < 0.05).

*Tremor evaluation using quality of life questionnaire (QUEST).* Upon analyzing the data from the initial pre-conversion visit, the patients included in the study reported a health status of 70% and an overall quality of life (QoL) of approximately 80% (Figure 4A). However, a couple of patients recorded a health status of 40% and an overall QoL 45%. In one of these cases, tremors impacted the head, voice, and both upper and lower extremities, which also affected sexual life. Most patients exhibited moderate tremor in the right hand/arm, with less intense tremor in the left hand/arm (Figure 4B). Tremor was also present in the lower limbs of 7 out of 10 patients. Concerning the impact of tremor on various life dimensions, 9 out of 10 patients reported that tremor significantly affected their physical activity, while its impact on communication and work was minimal or nonexistent (Figure 4C). After 3 months of conversion, patients were self-assessed using the QUEST. They reported an increase in their health status to 75% while the overall QoL remained around 80%. Notably, patients with poorer general condition showed an improved overall impression after the conversion (Figure 4A). Examination of the tremor’s location and severity revealed a general reduction was observed after tacrolimus conversion, particularly in the upper and lower limbs (Figure 4B). Additionally, the impact of tremor on hobbies and physical activity was significantly reduced (Figure 4C). The overall analysis of tremor severity (Figure 4D) and the impact of tremor on five dimensions (Communication, Work/Finance, Hobbies/Leisure, Physical, and Psychosocial) using the summary index (Figure 4E), revealed that a better tacrolimus bioavailability significantly reduced the tremor in our cohort of patients.

#### 2.3.3. Impact of Conversion on Tacrolimus Dose, Blood Levels, and AUC

**Figure 5:**
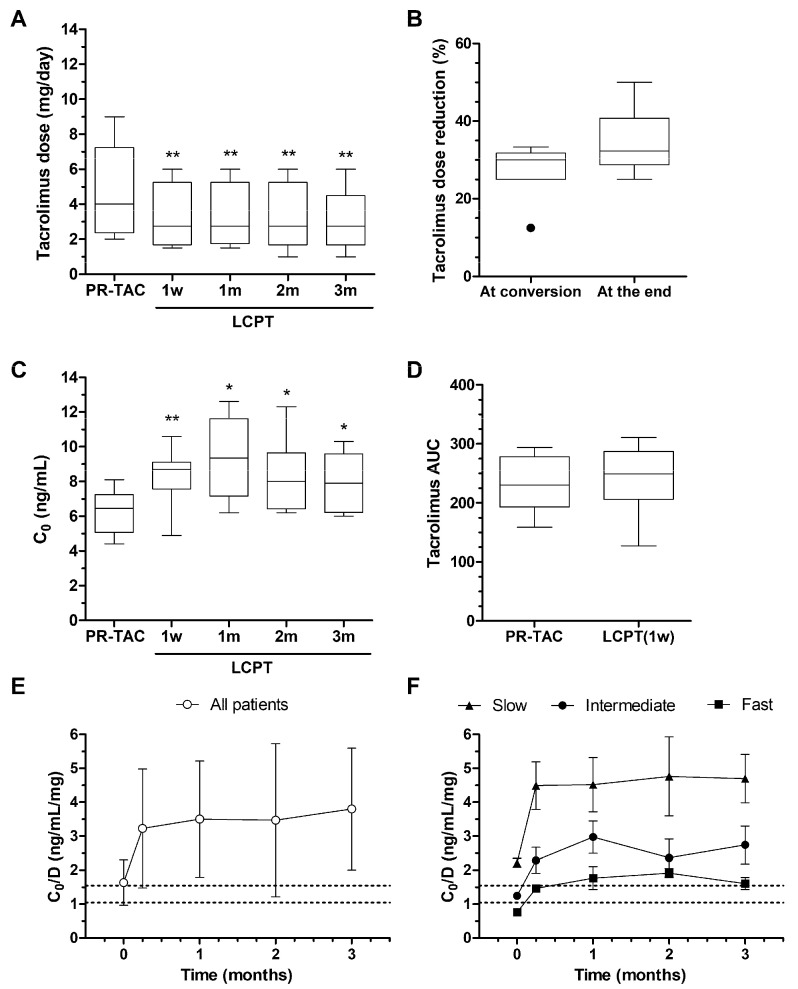
**Tacrolimus dose adjustments after conversion.** (**A**) Tacrolimus dose over the time of the study. (**B**) Tacrolimus reduction after conversion. (**C**) Tacrolimus trough levels (C_0_) over the study. (**D**) Tacrolimus AUC calculation when the patients were treated with PR-TAC and one week after conversion to LCPT. (**E**) Overall evolution of C_0_/D after conversion to LCPT. (**F**) Evolution of C_0_/D according to the type of metabolizer. Patients with a tacrolimus C_0_/D ratio < 1.05 ng/mL/mg were characterized as fast metabolizers, patients with a C_0_/D ratio of 1.05–1.54 ng/mL/mg as intermediate metabolizers, and those with a C_0_/D ratio ≥ 1.55 ng/mL/mg were defined as slow metabolizers. The dashed lines indicate C_0_/D ratio of 1.05 and 1.54ng/mL/mg. Significantly different when compared to PR-TAC (* *p* < 0.05; ** *p* < 0.01).

The median tacrolimus dose pre-conversion was 4.00 mg/day (95% CI; 2.834–6.466), that was reduced to 28.02% (95% CI; 23.60–32.45) at conversion. However, there were 4 patients that required an adjustment of tacrolimus dose related to clinical reasons; to reduce the tacrolimus trough levels (considered too high), an infection episode (overimmunosuppression), and incorporating the mTOR inhibitor instead of MMF. At the end of the study the mean tacrolimus dose was 3.075 mg/day (95% CI; 1.816–4.334), that was reduced 34.20% (95% CI; 28.54–39.87) respective to the pre-conversion dose (Figure 5).

Tacrolimus C_0_ levels increased in several patients after tacrolimus formulation conversion. Even after the adjustments, previously described, required in some patients, C_0_ was significantly higher after conversion (Figure 5C). There were no differences between simplified tacrolimus AUC for PR-TAC before conversion and LCPT formulation after one week of conversion (Figure 5D). However, it was not possible to assess Cmax, since only pre-dose, 2, 4, 6, and 24 h samples were collected. The reduction in tacrolimus dose after conversion modified the trough concentration/dose (C_0_/D) ratio (Figure 5E). According to the C_0_/D ratio, patients were categorized as slow, intermediate or fast metabolizers; the conversion from PR-TAC to LCPT recategorized slow and intermediate metabolizers to intermediate and slow metabolizers, respectively, (Figure 5F).

#### 2.3.4. Impact of Conversion on Correlations Between Tacrolimus AUC and Tremor AUC

**Figure 6:**
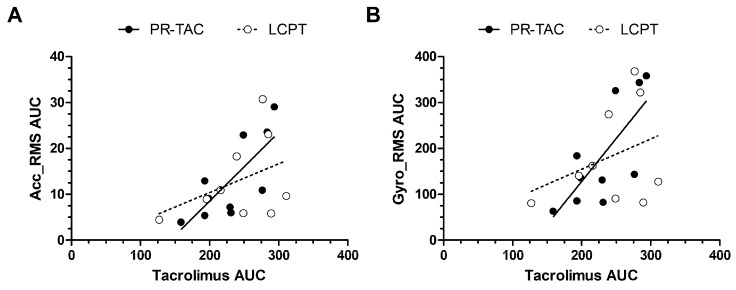
**Impact of conversion on Tremor AUC and tacrolimus AUC correlation.** (**A**) Accelerometer-RMS AUC and tacrolimus AUC correlation. (**B**) Gyroscope-RMS AUC and tacrolimus AUC correlation.

Tremor AUC, either Acc_RMS AUC or Gyro_RMS AUC, had a correlation with tacrolimus AUC in patients under PR-TAC treatment (r = 0.7455 and r = 0.7091, respectively, with *p* = 0.0174 and *p* = 0.0268) (Figure 6). The correlation was lost after the conversion to LCPT (r = 0.2667 and r = 0.150, respectively, with *p* = 0.493 and 0.708.)

### 2.4. Biomarkers

**Figure 7:**
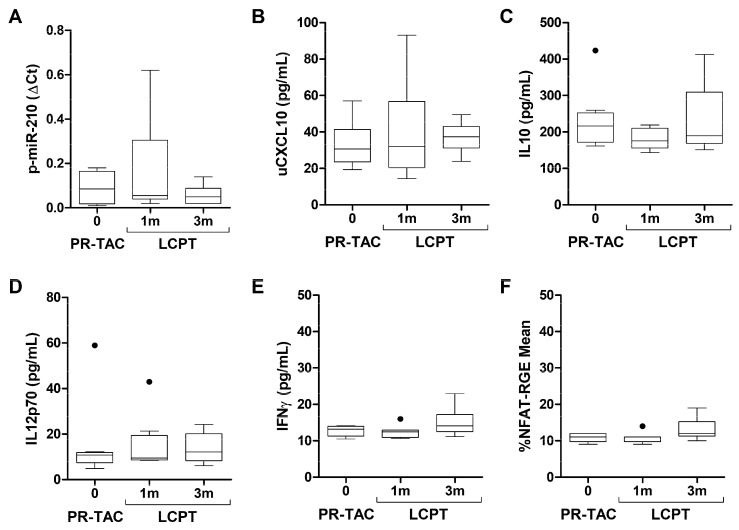
**Impact of conversion on immunological status biomarkers.** (**A**) Plasma concentration of miR-210. (**B**) Urinary concentration of CXCL10. (**C**) Plasma concentration of IL10. (**D**) Plasma concentration of IL12p70. (**E**) Plasma concentration of IFNγ. (**F**) Percentage of NFAT-RGE-mean. AUC, area under the curve; PR-TAC, prolonged release-tacrolimus; LCPT, extended-release tacrolimus; m, months.

We have evaluated several biomarkers related to the immunological status before and after tacrolimus conversion, including p-miR-210-3p, uCXCL10, IL10, IL12p70, IFNγ and the pharmacodynamic biomarker for tacrolimus, NFAT-RGE-mean (Figure 7). None of the biomarkers analyzed were modified after tacrolimus conversion. The levels of uCXCL10 remained below 100 pg/mL and NFAT-RGE-mean below 20%, in concordance with those observed in patients free of clinical events.

## 3. Discussion

Calcineurin is abundant in the brain, its inhibition could form the basis for the pathophysiology of neurotoxicity observed in patients on calcineurin inhibitor therapy. A number of mechanisms have been proposed to explain the neurological toxicity associated with calcineurin inhibitors. Calcineurin inhibitor neurotoxicity may be attributed to two major factors: the degree of exposure of neuronal tissue to the drugs and the effect of calcineurin inhibition on neuronal function [2,21,22,23]. Tremor is one of the most common neurotoxic side effects observed in recipients of solid organ transplantation under tacrolimus treatment. A tremor can be considered to have low (<4 Hz), medium (4–7 Hz) or high (>7 Hz) frequencies [24]. The screening study in our center showed that the recipients of kidney transplantation treated with PR-TAC had tremors with high frequencies (8.74 Hz). The frequency peak of around 8 Hz [18,25] was comparable to previous cohorts in liver and kidney transplant recipients treated with other calcineurin inhibitors (cyclosporin and IR-TAC). All studied patients had tacrolimus C_0_ within the targeted therapeutic range. In addition, Acc_RMS and Gyro_RMS parameters observed with the DyCare device allowed us to discriminate with higher accuracy patients with tremors. Tremor experienced by recipients under PR-TAC was not correlated with tacrolimus dose or blood levels, as in other studies [17,18]. In contrast, Riemersma et al. demonstrated that tacrolimus trough concentrations appeared as a main determinant of tremor among solid organ transplantation [26]. Furthermore, no correlation has been identified between the tremor levels and the time interval between drug administration and tremor measurement. Whilst certain studies have demonstrated an association between tremor and tacrolimus C_max_ [18,27], other research has indicated that tacrolimus exposure, whether measured by tacrolimus clearance or by dose-normalized AUC, is linked to extrarenal adverse effects in renal transplant recipients [28]. King et al. conducted a systematic review to determine if there was a correlation between tacrolimus exposure and new-onset tremor and concluded that more sensitive methods of determining tacrolimus exposure should be explored [29]. Interestingly, the present study revealed a correlation between the assessment of tremors at a time interval of 6 h, as determined by the calculation tremor AUC, and tacrolimus AUC, in patients under PR-TAC treatment. Following conversion to LCPT, this correlation was no longer evident (Figure 6).

In the conversion study, tremors were quantified using a DyCare device and self-evaluation using the Quality of Life in Essential Tremor Questionnaire (QUEST). Both strategies detected a reduction in tremor at the individual level; however, these changes were not significant until three months after conversion to LPCT, which may be attributed to the conversion process itself, as up to four of the ten patients required a greater reduction in the tacrolimus dose. This could be a key factor in explaining why the reduction in tremor was delayed. Furthermore, the patient cohort in the conversion study is not homogeneous; the range of tremor severity is broad, from very high to low levels. Consequently, the impact of the change in tacrolimus formulation may vary among patients receiving low doses of tacrolimus. Despite failing to provide a rationale for switching kidney transplant recipients from twice-daily tacrolimus formulation (IR-TAC) to once-daily tacrolimus formulations (PR-TAC or LCPT) with the aim of reducing intrapatient variability (IPV), the analysis of the occurrence of self-reported side effects observed a significant reduction in tremor in patients receiving LCPT [30].

To study the impact of the tacrolimus conversion on immunological status, we analyzed several biomarkers that have previously been related with rejection episodes or infections in solid organ transplant recipients.

Regarding the proposed pharmacodynamic biomarker for tacrolimus, Nuclear Factor of Activated T cell-Regulated Gene Expression (NFAT-RGE), all patients in our cohort presented an NFAT-RGE-mean of 11% (10.25–12.0%). We have previously shown that liver transplant recipients without rejection or infection exhibit a median NFAT-RGE-mean value of 14% (2–23%) [31]. In renal transplantation, Sommerer et al. demonstrated that patients with NFAT-RGE < 30% were free of rejection, while those with NFAT-RGE < 10% were at increased risk of infectious complications, particularly viral infections like CMV or BKV [32]. Our findings align with these observations, indicating that the patients in our cohort were clinically stable, without significant events such as rejection or infections. In addition, the median uCXCL-10 value observed in the present population was below the cut-off of 84.73 pg/mL that was previously established by our group for the risk of rejection (with 84% sensitivity and 80% specificity). This is consistent with patients who were free of rejection [33].

## 4. Methods

### 4.1. Patients and Procedures for the Screening Study

Kidney transplant recipients followed at Hospital Clínic Barcelona under treatment with PR-TAC and with reported symptoms of tremor were invited to participate in the study. All patients included had a minimum of one-year post-transplant with stable renal function and a minimum of three months on a stable dose of PR-TAC. Following the exclusion of potential neurological causes attributable to other medical conditions and pharmacological agents, a tremor analysis was conducted using the DyCare device. The following tests were performed on kidney transplant recipients and healthy volunteers. The tacrolimus dose and the time interval between the last tacrolimus dose and the assessment of tremor were recorded. Tremor-related parameters under consideration were Tremor Frequency (Hz), root-mean-square (RMS) Accelerometer (in degrees), and RMS Gyroscope (in degrees). In addition, fourteen kidney transplant recipients gave consent to participate in a transversal pilot study to establish a correlation between tremors using the DyCare device and tacrolimus blood concentration at different time points (Figure 1B).

### 4.2. Patients and Procedures from the Conversion Study

From the total number of patients who underwent tremor screening, ten patients gave the consent to participate in the conversion study. In this study, PR-TAC was switched to LCPT treatment following European public assessment reports (EPAR); LCPT maintenance dose should be 30% less than the IR-TAC or PR-TAC. The conversion study was conducted over 3 months following the schedule in Figure 1C. A tremor-related quality of life questionnaire (QUEST) was administered before the conversion and at the end of the study after three months following the conversion to assess the impact of tremors on their lives. The patients were also followed up to ascertain whether the change in medication impacted tremors with the DyCare device. Tremor measurements were performed before conversion on day 0, and then at 2 h, 4 h, 6 h, and 24 h after PR-TAC administration. After final tremor assessment and blood sampling to determine tacrolimus levels, the patient began LCPT treatment (day +1) of the protocol. At each time point, tremor was analyzed before tacrolimus administration and after 2, 4, 6, and 24 h (Figure 1C).

Tacrolimus AUC was performed before conversion and one week later. Tacrolimus trough blood samples were obtained before conversion, after 1 week, 1, 2, and 3 months to determine renal function and adverse event. The immunological status was assessed using a panel of biomarkers previously used in our group.

### 4.3. Tremor Quantification Using DyCare Device

Tremor was measured with a wearable wireless sensor DyCare provided by Bio-Sensing Solutions SL., Barcelona, Spain. The DyCare device is a high accuracy device with FCC and EC marks developed for motion capture, long-term data acquisition and real-time monitoring. It contains an Accelerometer, a Gyroscope and a magnetic sensor. The device is positioned on the fingers (Figure S1A). The tremor measurement protocol was described in the Supplementary Data section. The acquired data was transferred via Bluetooth to the PC terminal, where it was stored and processed. Subsequently to the acquisition, the software provided by DyCare generated data sets containing Tremor Frequency, Accelerometer root mean square (Acc_RMS), and Gyroscope root mean square (Gyro_RMS) (Figure S1B,C). The root mean square (RMS) frequency of a spectrum is a single number that represents the overall energy level in each frequency range.

In addition, we have calculated the area under the curve (AUC) of tremors using Acc_RMS or Gyro_RMS evaluated before tacrolimus administration and after 2, 4, 6, and 24 h. Acc_RMS AUC and Gyro_RMS AUC were calculated at different time points; before conversion, after 1 week and after 1, 2, and 3 months.

### 4.4. Quality of Life Measures

The Quality of Life in Essential Tremor Questionnaire (QUEST). This contains two scales (from 0 to 100; 20 steps) for self-evaluation of General Health Status and Global Quality of Life, with questions addressing Essential Tremor (ET)-related impact on sexual satisfaction, treatment (side effects and satisfaction), and job situation. It also includes a self-assessment of tremor presence (hours per day with tremors in any part of the body) and tremor severity (in the head, voice, and both arms and legs) scored from 0 (never) to 4 (severe). In this study, a total of that tremor severity scale was obtained by summing the six items’ scores (range: 0–24) to obtain a global index of perceived tremor severity to be compared and correlated with other variables in the study. The questionnaire, which was designed to assess HRQoL associated with ET, consists of 30 items divided into five dimensions (Communication, Work/Finance, Hobbies/Leisure, Physical, and Psychosocial). Each item is scored from 0 (never) to 4 (always), and each dimension scored is expressed as a percentage of the maximum possible score. A summary index, representing the mean of the five dimensions scores, can be calculated [34]. The Spanish (Castilian) version was generated by Martinez-Martin et al. and provided by movementdisorders.org [35].

### 4.5. Blood Sample Collection

Blood samples were collected from patients included in the screening and conversion studies to investigate correlations between tacrolimus blood concentrations and tremor. In addition, patients included in the conversion study were followed over 3 months after conversion to determine the impact of tacrolimus conversion regarding renal function and the immunosuppressive status [9].

All determinations of the changes that occur in the biomarkers to evaluate the evolution of the graft as well as the monitoring of the immunosuppressive treatment were carried out entirely in the Pharmacology and Toxicology Laboratory (CDB) of the Hospital Clínic of Barcelona.

### 4.6. Tacrolimus AUC

Whole-blood tacrolimus concentrations were measured via the tacrolimus-CMIA-Architect assay (Abbott, Wiesbaden, Germany) according to the manufacturer’s instructions. Fresh, unfrozen samples were analyzed daily. The laboratory’s participation in the United Kingdom External Analytical Quality Assessment Service ensured compliance with LGC Standard Proficiency Testing.

We measured the tacrolimus area under the curve (AUC) the day before conversion and one week after conversion. On each day, we collected blood samples before drug administration, after 2, 4, 6, and 24 h. According to previous studies [4], we calculated abbreviated AUC by each tacrolimus formulation (Supplementary Data):

PR-TAC AUC (0H, 4H, 6H) = 24.59 + 10.13×Tac-0H + 8.02×Tac-6H + 3.42×Tac-4H

LCPT AUC (2H, 6H, 24H) = −0.408 + 10.015×Tac-6H + 9.399×Tac-24H + 3.536×Tac-2H

### 4.7. Biomarkers Analysis

#### 4.7.1. Plasmatic Expression of miRNA-210-3p

At the time of the clinical visits, plasmatic miR-210-3p expression was assessed by quantitative real-time PCR (qPCR) using a LightCycler 480 Real-Time PCR System (Roche, Basel, Switzerland). Blood samples (3 mL) were collected into EDTA-K3 tubes at each visit according to the study design, prior to the immunosuppressant administration (pre-dose). After centrifugation at 3000 rpm for 10 min, plasma was collected and stored in RNase-free tubes at −70 °C for batched analysis.

Plasmatic expression was analyzed as previously described by our group [36]. Briefly, total RNA was purified from patient plasma according to the manufacturer’s instructions (miRCURY™ RNA Isolation Kits—Biofluids from Qiagen, Hilden, Germany) and reverse transcribed into cDNA. qPCR was performed using a miRCURY LNA SYBR Green PCR Kit (Qiagen ID: 339347), Polyadenylation, and cDNA Synthesis System (Qiagen, Hilden, Germany). The amplification curves were analyzed using Roche LC Software for determining Cq by the second derivative method. ∆Cq was calculated as the difference in Cq values between the miRNA target and the reference control (miR-103a-3p and miR-191-5p), following the manufacturer’s instructions; relative expression levels of target miRNAs were then evaluated within a sample according to the formula 2^−∆Cq^, where high values corresponded to higher expression.

#### 4.7.2. Urinary CXCL-10 Analysis

For the analysis of urinary CXCL-10 at the time of the clinical visits, urine samples were centrifuged at 3000 rpm during 10 min and supernatant was stored at −70 °C for batched analysis. Concentrations of CXCL-10 (pg/mL) were measured by ELISA kit (Quantikine ELISA human CXCL-10/IP10, R&D Systems, Minneapolis, MN, USA), according to the manufacturer’s instructions. The minimum detectable concentration of CXCL-10 was 1.67 pg/mL.

#### 4.7.3. Soluble Cytokine Production

Sodium heparin anticoagulated whole blood samples were collected at the time of clinical visits prior to the immunosuppressant administration (pre-dose). Soluble cytokine production was tested in the supernatants after 24 h of culture for IL-12p70 and after 72 h for IL-10. IFNγ production was analyzed in plasma samples. Concentrations were measured by ELISA (R&D Systems, Minneapolis, MN, USA).

For IL-12p70 and IL-10, patient blood was diluted 1:10 in RPMI-1640 culture medium and 10% FCS. Diluted blood (150 μL) was added to the wells of flat-bottom 96-well tissue culture microtiter plates (12 wells/sample). Then, 50 μL of LPS (100 ng/mL) was added to each well to stimulate IL-12-monocyte production and 50 μL of ConA (15 μg/mL) was added to each well to stimulate IL-10 production. After 24 h or 72 h of incubation at 37 °C in a 5% CO2 incubator, the plates were centrifuged, and the supernatants of 12-wells were pooled and frozen at −70 °C until assayed by ELISA.

For plasmatic IFNγ analysis, blood samples were collected and centrifugated at 3000 rpm for 10 min at room temperature. Plasma was collected and stored at −70 °C for batch analysis.

The sensitivity of IL-measurement was less than 0.2 pg/mL for IL-12p70, and less than 5 pg/mL for IL-10 and IFNγ.

#### 4.7.4. NFAT-Regulated Gene Expression

NFAT-RGE was evaluated as previously described by our group following on the method described by Giese et al. [31,37].

*Sample preparation.* Residual NFAT-RGE was assessed in whole blood samples. Heparinized peripheral blood (pre-administration and 2 h after PR-TAC administration or 5 h after LCPT administration) was stimulated with PMA(100 ng/mL) and ionomycin (5 ng/mL) (SIGMA-Aldrich Corp, St Louis, MO, USA) for 3 h at 37 °C and 5% CO_2_. After red cells were lysed with ACK lysis buffer, leukocytes were lysed with 700 μL of MagNA-Pure lysis buffer supplemented with an additional 1% (*w*/*v*) DTT (Roche, Mannheim, Germany), and the sample was frozen at −70 °C. mRNA was isolated with the Magna-Pure-LC device using the mRNA standard protocol for cells. RNA was reverse transcribed in a thermocycler using AMV reverse transcriptase and oligo (dT) as a primer (First Strand cDNA Synthesis kit; SIGMA-Aldrich Corp, St Louis, MO, USA) according to the manufacturer’s instructions. After cDNA synthesis, the reaction mix was diluted to a final volume of 200 μL, and stored at −20 °C until PCR analysis.

#### 4.7.5. Quantitative Analysis of IL-2, IFNγ and GM-CSF Gene Expressions

Gene expression was quantified using quantitative real-time PCR (qPCR) with an LC480 Light Cycler (Roche, Mannheim, Germany). Target sequences were amplified using commercially available LightCycler Primer sets (Search-LC, Heidelberg, Germany) with the Light Cycler FastStart DNA SYBR Green I kit (Roche, Mannheim, Germany) according to the manufacturer’s protocol. mRNA input was normalized by the constant expression value of two housekeeping genes (β-actin and cyclophilin B). Quantification cycle values were determined by the fit point method and a baseline of −0.6. RGE after tacrolimus administration was calculated as C_peak_/C_0_ × 100, where C_0_ is the adjusted number of transcripts before tacrolimus administration and C_peak_ is the number of transcripts 2 h after PR-TAC administration or 5 h after LCPT administration. The results were expressed as NFAT-RGE-mean that was calculated as mean RGE from the three measured NFAT-regulated genes: IL-2, IFNγ, and GM-CSF.

### 4.8. Statistics

Descriptive statistics were summarized as mean with SD, minimum, maximum, and median with a 95% confidence interval (CI). All statistical tests were carried out at a two-side, 5% significance level. The Wilcoxon test, a nonparametric statistical test that compares two paired groups, was used. Spearman’s was used to analyze the correlation between tacrolimus blood levels and tremor parameters.

## 5. Limitations

The present conversion study was conducted with a limited number of patients. The results suggest that patients treated with PR-TAC who experience tremors may benefit from conversion to LCPT. However, further studies with larger patient cohorts or even multicenter trials should be conducted to demonstrate the benefit of the conversion. This is particularly necessary in view of the inter- and intraindividual variability in tacrolimus pharmacokinetics. Although the C_0_/D ratio was assessed, we do not dispose with the pharmacogenetic polymorphism information of the patients.

## 6. Conclusions

This study demonstrates that a change to a more beneficial tremor profile is evident in the context of conversion from PR-TAC to LCPT. Furthermore, in spite of the significant dose reduction, all conversion patients maintained similar trough concentrations and showed no impact on renal function or immunological status. The DyCare device facilitates precise tremor quantification in kidney transplant recipients, supporting physicians in optimizing treatments strategies, such as adjusting tacrolimus doses or transitioning to formulations with improved bioavailability.

## Figures and Tables

**Table 1:** Measurement of tremors using the DyCare device in kidney transplant recipients and healthy volunteers (Observational Screening study).

	Healthy Volunteers	KTx Recipients	*p* Value
Number	12	70	
Tacrolimus dose (mg/day)	0	4.84 (4.15–5.53)	
Time after last dose (h)	NA	6.48 (5.15–7.82)	
Frequency (Hz)	4.87 ± 1.06	8.74 ± 0.11	0.0163
Accelerometer RMS (×100)°	0.54 ± 0.05	1.36 ± 0.08	<0.001
Gyroscope RMS (×100)°	5.57 ± 0.46	17.38 ± 1.16	<0.001

**Table 2:** **Blood parameters from patients included in the conversion study.** GFR, glomerular filtrate rate; AST, aspartate amino transferase; ALT, alanine amino transferase. Mann–Whitney tests were performed.

Blood Parameters	Baseline	At the End	Significance
Creatinine mg/dL	1.51 ± 0.13	1.68 ± 0.11	ns
eGFR ml/min/1.73 m^2^	49.9 ± 5.97	41.0 ± 4.33	ns
Na mEq/L	141.5 ± 4.2	143.4 ± 1.6	ns
K mEq/L	4.24 ± 0.13	4.40 ± 0.12	ns
Glucose	100.5 ± 9.46	97.4 ± 6.34	ns
AST U/L	20.10 ± 1.92	18.88 ± 2.54	ns
ALT U/L	20.90 ± 2.51	22.50 ± 3.21	ns
Total Cholesterol	178.4 ± 14.35	166.4 ± 14.40	ns

## Data Availability

The original contributions presented in this study are included in the article/Supplementary Materials. Further inquiries can be directed to the corresponding author(s).

## Supplementary Materials

The following supporting information can be downloaded at: https://www.mdpi.com/article/10.3390/ph18101488/s1, Supplementary methods. Tremor measurement protocol using DyCare device. Tacrolimus area under the curve (AUC). Supplementary table. Table S1. Descriptive data for each biomarker analyzed at different time points. Supplementary figures. Figure S1. DyCare device information. Figure S2. ROC curve tremor parameters. Figure S3. Impact of tacrolimus blood levels on tremor parameters: Acc_RMS (A) and Gyro_RMS (B).
