# Supplementary material for: Tacrolimus-Associated Tremor in Renal Transplant Patients: Potential Impact of the Galenic Formulation
Source: Pharmaceuticals (Basel). 2025 Oct 3;18(10):1488. doi: 10.3390/ph18101488 (PMC12566630; doi:10.3390/ph18101488)

Tacrolimus-associated tremor in renal transplant patients: Potential impact of the galenic formulation

Jordi Rovira[1,2], Olga Millán[3,4], Pedro Ventura-Aguiar[1,2,5],Mercè Brunet[3,4,*], Fritz Diekmann[1,2,5,*]

[1]Nephrology and Transplantation (LENIT), IDIBAPS, Barcelona, Spain.

[2]RICORS(RD21_0005_0003 -Next Generation). Instituto de Salud Carlos III, Spain.

[3]Pharmacology and Toxicology Section, CDB, IDIBAPS, Hospital Clinic of Barcelona, University of Barcelona, Spain.

[4]Biomedical Research Center in Hepatic and Digestive Diseases (CIBERehd), Instituto de Salud Carlos III, Spain.

[5]Department of Nephrology and Kidney Transplantation, ICNU, Hospital Clínic de Barcelona, Spain.

*These authors contributed equally to this study

SUPPLEMENTARY DATA

Supplementary methods

Tremor measurement protocol using Dycare device

It is imperative that the individual refrain from any form of verbal expression, coughing, or movement during the measurement.

The device sensor was affixed using a bio-adhesive substance attached to the index and middle fingers.

The patient should be seated in an upright position with the arm extended and the hand raised to shoulder height. If this is not possible, it is proposed that the arm be extended to a position at an angle of 30° with the hand at shoulder height. The subject should remain focused on a fixed point for a period of 30 seconds. It is proposed that measurements of at least 30 seconds per patient be taken, with a minimum of 15 seconds of useful signal extracted. We repeated the measurement 2-3 times in order to obtain a correct measurement.

Tacrolimus area under the cureve (AUC)

The following requirements must be met to undertake a step-by-step multiple linear regression analysis:

Determination coefficient of the regression line >0.80.

Normality of the residuals of the ANOVA

Shapiro-Wilks (S-W) test with p > 0.05.

Independence of the errors.

Durbi-Watson (DW) test.

Statistic comprised between 2 and 3.

Homogeneity of variance of the regression study. The Breusch-Pagan (BP) test and the Lagrange multipier (LP) statistic with p> 0.05 are also to be considered.

It is also vital to ensure that there is no multicollinearity, and that the variance inflation factos (VIF) are less than 10.

The formula error prediction requirements are as follows:

Standard error of the relative mean (ME) < 5%.

Standard error of the absolute mean (MAE) < 10%.

Ratio of the root mean squared error (RMSE) < 15%.

PR-TAC AUC (0H, 4H, 6H) = 24,59 + 10,13*Tac-0H + 8,02*Tac-6H + 3,42*Tac-4H

$r2 = 0,897$

S-W; p= 0,417

DW = 2,495

BP; p=0,323

VIF = 6,701; 3,214; 7,914

ME = 0,097 %

MAE = 6,79 %

RMSE= 9,26 %

LCPT AUC (2H, 6H, 24H) = -0,408 + 10,015*Tac-6H + 9,399*Tac-4H + 3,536*Tac-2H

$r2 = 0,990$

S-W; p= 0,858

DW = 2,091

BP; p=0,235

VIF = 4,503; 1,943; 5,434

ME = 0,031 %

MAE = 0,983 %

RMSE= 1,296 %

Supplementary Table

Table S1. Descriptive data for each biomarker analyzed at different time points.

**Visit 0 – PR-TAC (Pre-conversion)**	Plasma miR-210	uCXCL-10 (pg/mL)	IL-10 (pg/ml)	IL-12p70 (pg/ml)	IFNγ (pg/ml)	%RGE Mean
**Mean**	0.157	29.99	222.4	13.90	12.83	10.90
**Median**	0.106	29.11	216.3	10.08	13.03	11.00
**SD**	0.206	12.59	80.63	16.01	1.296	1.197
**Min.**	.01	17.60	133.4	4.87	10.46	9.00
**Max.**	0.72	57.06	423.5	58.95	14.09	12.00
**Visit 2 – LCPT (1 month)**	Plasma miR-210	uCXCL-10 (pg/mL)	IL-10 (pg/ml)	IL-12p70 (pg/ml)	IFNγ (pg/ml)	%RGE Mean
**Mean**	0.168	41.47	183.4	13.67	12.20	10.80
**Median**	0.071	32.01	175.8	9.32	12.26	11.00
**SD**	0.197	26.66	51.25	11.20	1.706	1.398
**Min.**	0.02	14.46	105.6	4.99	9.96	9.00
**Max.**	0.62	93.10	293.0	42.99	16.04	14.00
**Visit 4 – LCPT (3 months)**	Plasma miR-210	uCXCL-10 (pg/mL)	IL-10 (pg/ml)	IL-12p70 (pg/ml)	IFNγ (pg/ml)	%RGE Mean
**Mean**	0.060	37.42	229.1	13.49	16.14	13.12
**Median**	0.052	37.37	189.8	12.17	14.62	12.00
**SD**	0.041	8.007	95.19	6.587	4.464	2.949
**Min.**	0.02	23.87	151.2	6.16	11.15	10.00
**Max.**	0.14	49.48	412.6	24.32	22.97	19.00

Supplementary Figures

Figure S1. Dycare device information. (A) Dycare device localization and specifications. (B) Accelerometer information provided by DyCare software. (C) Gyroscope information provided by DyCare software.

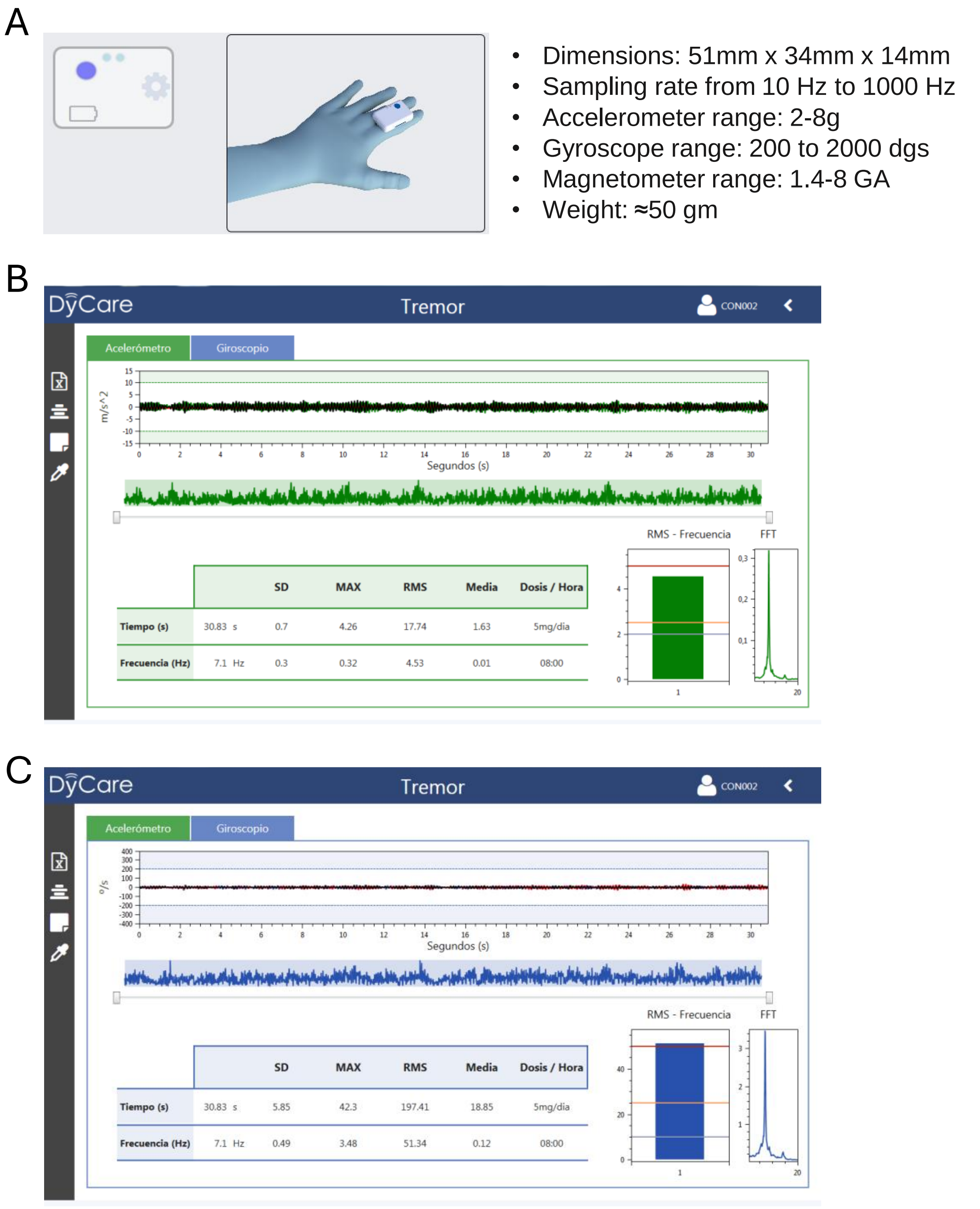

Figure S2. ROC curve tremor parameters. (A) ROC curve of tremor frequency. (B) ROC curve of Accelerometer_RMS. (C) ROC curve of Gyroscope_RMS. (D) Data from ROC curves determined.

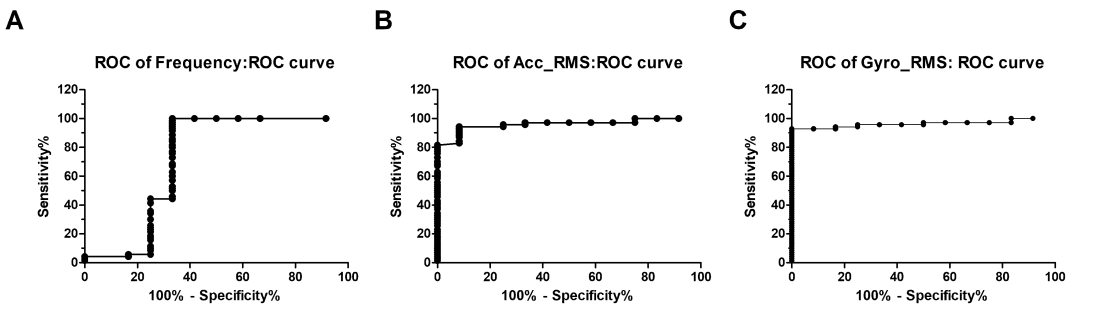

Area under the ROC curve	Frequency	Acc_RMS	Gyro_RMS
Area±SD	0.7155±0.1201	0.9601±0.02125	0.9631±0.01971
95% confidence interval	0.4801 to 0.9509	0.9185 to 1.002	0.9245 to 1.002
P value	0.01761	< 0.0001	< 0.0001

Figure S3. Impact of tacrolimus blood levels on tremor parameters: Acc_RMS (A) and Gyro_RMS (B).

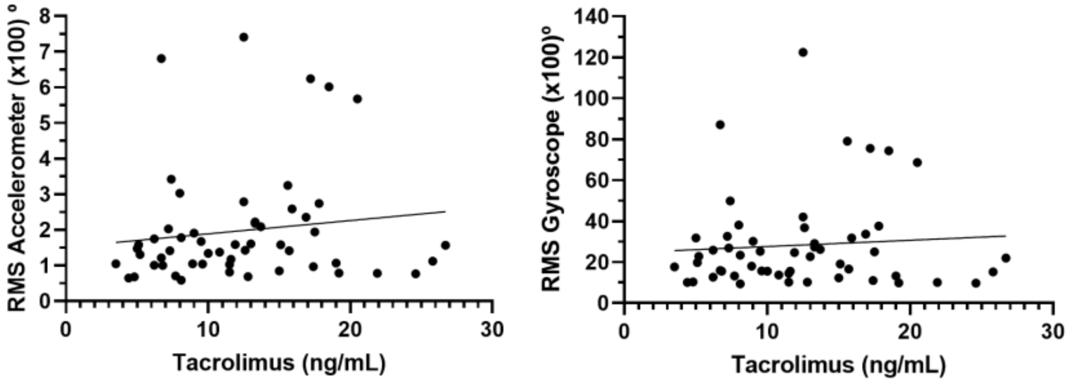

Supplement: Supplementary file 1 [file pharmaceuticals-18-01488-s001.zip › pharmaceuticals-3805039-supplementary.pdf]
